# On Iodine Clysters in the Treatment of Dysentery

**Published:** 1852-07

**Authors:** 


					﻿From Henle’s Zeitschrift, band. x. p. 338.
On Iodine Clysters in the treatment of Dysentery. By Dr. Eimer.
Dr. Eimer believes that the great point to which practitioners
have to direct their attention, is the enormous amount of organic
losses consequent on the continuance of this affection—-so that,
according to (Esterlen,* within three weeks, more than the entire
blood-mass may pass away as albumen in the stools. As a means
of cutting these discharges short, he strongly recommends iodine
clysters ; which, in recent cases, may at once arrest the progress
of the disease, and in all diminish the number of stools, and nor-
malize their condition, whatever the individual peculiarities of the
case may be. From five to ten grains of iodine, and as much iod.
pot., are administered in two or three ounces of water, from two
to four times a day—twice daily usually sufficing. If the rectum
is too irritable to retain it, ten or fifteen drops of tr. opii are to be
added, and a mucilaginous vehicle substituted for water. In spite
of unfavorable conditions, so constantly successful did Dr. Eimer
find this remedy during an epidemic, that he believes the disease
will, as a general rule, bo found curable by it, if it be resorted
to before the organic changes in the intestine have advanced too
far, exhaustion becomes too considerable, or more important com-
plications set up. In some slight cases it was employed alone.
Generally, a simple oily emulsion was also administered, and some-
times acetate of lead and opium.
* See British and Foreign Medico-Chirurgical Review. vol. v. p 245.
				

